# PPARα downregulates airway inflammation induced by lipopolysaccharide in the mouse

**DOI:** 10.1186/1465-9921-6-91

**Published:** 2005-08-09

**Authors:** Carine Delayre-Orthez, Julien Becker, Isabelle Guenon, Vincent Lagente, Johan Auwerx, Nelly Frossard, Françoise Pons

**Affiliations:** 1 EA 3771, Inflammation et environnement dans l'asthme, Faculté de Pharmacie, Université Louis Pasteur-Strasbourg I, Illkirch, France; 2 INSERM U620, Faculté des Sciences Pharmaceutiques, Université de Rennes 1, Rennes, France; 3 Institut de Génétique et de Biologie Moléculaire et Cellulaire, CNRS/Inserm/ULP, Illkirch, France

**Keywords:** PPARα, lipopolysaccharide, inflammation, neutrophil, macrophage, matrix metalloproteinase, mouse

## Abstract

**Background:**

Inflammation is a hallmark of acute lung injury and chronic airway diseases. In chronic airway diseases, it is associated with profound tissue remodeling. Peroxisome proliferator-activated receptor-α (PPARα) is a ligand-activated transcription factor, that belongs to the nuclear receptor family. Agonists for PPARα have been recently shown to reduce lipopolysaccharide (LPS)- and cytokine-induced secretion of matrix metalloproteinase-9 (MMP-9) in human monocytes and rat mesangial cells, suggesting that PPARα may play a beneficial role in inflammation and tissue remodeling.

**Methods:**

We have investigated the role of PPARα in a mouse model of LPS-induced airway inflammation characterized by neutrophil and macrophage infiltration, by production of the chemoattractants, tumor necrosis factor-α (TNF-α), keratinocyte derived-chemokine (KC), macrophage inflammatory protein-2 (MIP-2) and monocyte chemoattractant protein-1 (MCP-1), and by increased MMP-2 and MMP-9 activity in bronchoalveolar lavage fluid (BALF). The role of PPARα in this model was studied using both PPARα-deficient mice and mice treated with the PPARα activator, fenofibrate.

**Results:**

Upon intranasal exposure to LPS, PPARα^-/- ^mice exhibited greater neutrophil and macrophage number in BALF, as well as increased levels of TNF-α, KC, MIP-2 and MCP-1, when compared to PPARα^+/+ ^mice. PPARα^-/- ^mice also displayed enhanced MMP-9 activity. Conversely, fenofibrate (0.15 to 15 mg/day) dose-dependently reduced the increase in neutrophil and macrophage number induced by LPS in wild-type mice. In animals treated with 15 mg/day fenofibrate, this effect was associated with a reduction in TNF-α, KC, MIP-2 and MCP-1 levels, as well as in MMP-2 and MMP-9 activity. PPARα^-/- ^mice treated with 15 mg/day fenofibrate failed to exhibit decreased airway inflammatory cell infiltrate, demonstrating that PPARα mediates the anti-inflammatory effect of fenofibrate.

**Conclusion:**

Using both genetic and pharmacological approaches, our data clearly show that PPARα downregulates cell infiltration, chemoattractant production and enhanced MMP activity triggered by LPS in mouse lung. This suggests that PPARα activation may have a beneficial effect in acute or chronic inflammatory airway disorders involving neutrophils and macrophages.

## Background

Inflammation is a feature of both acute lung injury and chronic airway diseases. In chronic airway diseases such as chronic obstructive pulmonary disease (COPD), it is associated with profound tissue remodeling that contributes to impaired lung function [1]. Lipopolysaccharides (LPS), which are biological active components of the outer membrane of gram-negative bacteria, are important inducers of lung inflammation. Inflammatory response triggered by LPS is characterized by neutrophil and macrophage recruitment and by the release of chemoattractants including tumor necrosis factor-α (TNF-α), and the CXC and CC chemokines, interleukin-8 (IL-8) and monocyte chemoattractant protein-1 (MCP-1), respectively [2-5]. These inflammatory events reproduce some of the features of the inflammatory response observed during acute lung injury or COPD [1,6].

In mice, airway inflammation induced by LPS is associated with an increase of the matrix metalloproteinases (MMP), MMP-2 and MMP-9 [7,8]. MMP are a family of zinc- and calcium-dependent endopeptidases that play a major role in tissue remodeling [9,10]. Indeed, MMP degrade the majority of the extracellular matrix (ECM) proteins, including collagens, gelatins and proteoglycans, an activity which may contribute to lung injury by promoting infiltration accross basement membrane and activation of inflammatory cells [9,11]. Among MMP, MMP-2 (gelatinase A) preferentially produced by fibroblasts and other connective tissue cells, and MMP-9 (gelatinase B) mainly found in inflammatory cells, such as neutrophils and macrophages are of particular interest, since they cleave the major constituent of basement membrane, type IV collagen [9,10].

With the exception of neutrophils, normal tissues do not store MMP and constitutive expression is minimal. However, during inflammation and tissue remodeling, MMP expression is upregulated [9]. Levels or activity of several MMP have been found to be raised in animal models of acute lung injury (for review: [12]). Upregulation of MMP was also observed in chronic airway diseases associated with tissue remodeling, such as asthma and COPD (for review: [1,13]). Indeed, increased levels of MMP-9 have been reported in bronchoalveolar lavage fluid (BALF), blood or sputum from patients with asthma or COPD [14-17].

Peroxisome proliferator-activated receptor-α (PPARα) is a ligand-activated transcription factor, that belongs to the nuclear receptor family. PPARα regulates gene expression by binding as a heterodimeric complex with the retinoid X receptor to specific DNA sequences known as peroxisome proliferator response elements. PPARα was first identified for its role in the regulation of lipid and carbohydrate metabolism (for reviews: [18,19]). However, subsequent data have demonstrated that it exhibits also a potent anti-inflammatory activity. Indeed, mice deficient in PPARα (PPARα^-/-^) were reported to display an exacerbated reaction to various inflammatory stimuli, including LPS in the skin and the vessel [20-22]. Conversely, animals treated with PPARα activators such as fibrates exhibited a decreased response. Anti-inflammatory activity of fibrates appeared as unrelated to their lipid-lowering activity, since treatment with fenofibrate was shown to reduce inflammatory response associated with cerebral injury in absence of any improvement in plasma lipid levels in the mouse [23]. More recently, PPARα agonists were shown to reduce LPS- and cytokine-induced MMP-9 secretion in human monocytes and rat mesangial cells, suggesting that PPARα may also play a beneficial role in tissue remodeling [24,25].

We have here investigated the role of PPARα in a mouse model of LPS-induced airway inflammation characterized by cell infiltration, production of chemoattractants and increased MMP activity. This study was undertaken using both PPARα-deficient mice and mice treated with the PPARα activator, fenofibrate.

## Materials and methods

### Animals

Male wild-type (PPARα^+/+^) and homozygous knockout (PPARα^-/-^) mice (SV/129/C57BL/6) were expanded from breeding pairs [26] and used at the age of 9 weeks. Nine-week-old male C57BL/6 mice were purchased from Charles River Laboratories (Saint-Germain-sur-l'Arbresle, France). Animals were maintained under controlled environmental conditions with a 12 h/12 h light/dark cycle according to the EU guide for use of laboratory animals. Food (UAR-Alimentation, Villemoisson, France) and tap water were available ad libitum. Animal experimentation was conducted with the approval of the government body that regulates animal research in France.

### LPS administration

LPS (*Escherichia coli*, serotype 055:B5, Sigma Chemical, Saint Quentin Fallavier, France) prepared in saline was administered by i.n. instillation for 4 consecutive days at the dose of 40 μg/kg. Control animals received saline instead of LPS. Instillations (12.5 μl per nostril) were carried out under anaesthesia (50 mg/kg ketamine and 3.33 mg/kg xylazine given i.p.).

### Treatment with fenofibrate

Fenofibrate (Sigma Chemical) suspended in 1% carboxymethylcellulose (low viscosity, Sigma) in water was administered per os once daily for 10 days at increasing doses (0.15 to 15 mg/day), as previously described [27]. Duration of treatment was selected from a previous study showing protection against myocardial injury in mice [28]. Control animals received equivalent volumes (100 μl) of 1% carboxymethylcellulose (CMC) in similar conditions.

### Collection of bronchoalveolar lavage fluids

Eighteen to twenty-four hours after the last LPS administration, mice were anaesthetized by i.p. injection of ketamine (150 mg/kg) and xylazine (10 mg/kg). A plastic cannula was inserted into the trachea and airways were lavaged by 10 instillations of 0.5 ml ice-cold saline supplemented with 2.6 mM EDTA (saline-EDTA). BALF recovered from the two first instillations were centrifuged (4100 rpm for 5 min at 4°C), and the resulting supernatant was stored at -20°C until MMP and cytokine measurements.

### Determination of total and differential cell counts

BALF were centrifuged (1200 rpm for 5 min at 4°C) to pellet cells and erythrocytes were lysed by hypotonic shock. Cells were then resuspended in 500 μl ice-cold saline-EDTA and total cell counts were determined using a hemocytometer (Neubauer's chamber). Differential cell counts were assessed on cytologic preparations obtained by cytocentrifugation (Cytospin 3, Shandon Ltd, Runcorn, Chershire, UK) of 200 μl of diluted BALF (250 000 cells/ml in ice-cold saline-EDTA). Slides were stained with Hemacolor (Merck, Dormstadt, Germany) and determinations were performed by counting at least 400 cells for each preparation. Cells were identified as macrophages and neutrophils, and expressed as absolute numbers from total cell counts.

### Determination of cytokine and chemokine levels

Tumor necrosis factor-α (TNF-α), keratinocyte derived-chemokine (KC), macrophage inflammatory protein-2 (MIP-2) and monocyte chemoattractant protein-1 (MCP-1) were quantified in BALF using capture ELISA kits according to instructions provided by the manufacturers (PharMingen for TNF-α and R&D Systems Europe (Lille, France) for KC, MIP-2 and MCP-1).

### Gelatin zymography for determination of gelatinase activity

BALF samples were separated under non-reducing conditions by electrophoresis on a 7% acrylamide-separating gel containing 1 mg/ml gelatin and sodium dodecyl sulfate, as previously described [7]. After electrophoresis, gels were washed twice with 2.5% Triton X-100, rinsed with water and incubated overnight at 37°C in 50 mM Tris pH 8.0 containing 5 mM CaCl_2 _and 1 nM ZnCl_2_. Gels were stained with Coomassie Brilliant blue and destained in a 25% ethanol and 10% acetic acid solution. Gelatinase (MMP-2 and MMP-9) activities that appeared as clear bands against a blue background were quantified by measuring intensity of the bands by densitometry using the Densylab software (Bioprobe Systems, Les Ulis, France). Results were expressed as percentages of the intensity of a given sample loaded as internal standard onto each gel.

### Histology

Lungs were perfused *in situ*, collected and immersed in 4% paraformaldehyde for 24 h at 4°C. Fixed lungs were rinsed in phosphate-buffered saline, dehydrated and embedded in paraffin using standard procedures. Five-micrometer tissue sections were stained with hematoxylin-eosin and observed under light microscopy.

### Statistical analysis

Data are presented as means ± SEM. Statistical differences were analyzed from raw data by analysis of variance followed by unpaired two-tailed Student's t-test with a Bonferroni correction.

## Results

### Increased cell infiltration, chemoattractant production and MMP activity in PPARα^-/- ^mice upon exposure to LPS

**Figure 1:**
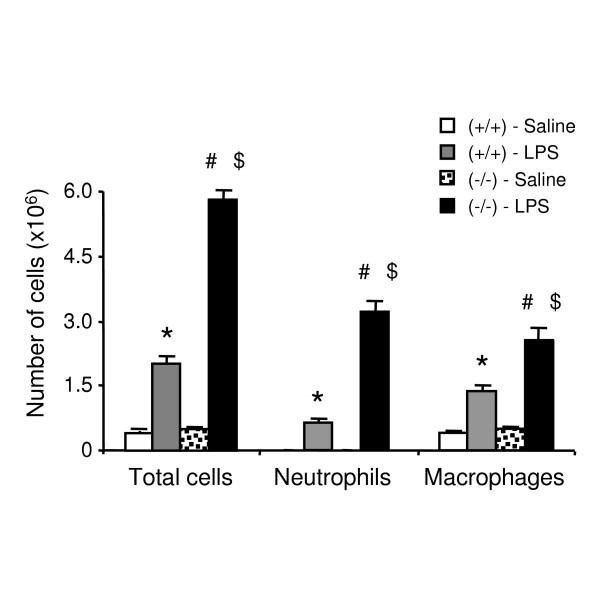
**Number of total cells, neutrophils and macrophages in BALF from PPARα^+/+^(+/+) and PPARα^-/- ^(-/-) mice exposed to LPS or saline**. Data are mean ± SEM of n = 10–13 animals. Statistically significant differences at α = 0.05: (*) when compared to PPARα^+/+ ^mice treated with saline; (#) when compared to PPARα^-/- ^mice treated with saline; and ($) when compared to PPARα^+/+ ^mice treated with LPS.

Saline-exposed PPARα^-/- ^mice exhibited no differences in total cell and macrophage count in BALF when compared to saline-exposed PPARα^+/+ ^animals (Figure 1). Upon exposure to LPS, both PPARα^+/+ ^and PPARα^-/- ^mice displayed a significant increase in total cell, neutrophil and macrophage number, when compared to animals exposed to saline (Figure 1). However, these increases were 2.9- (p < 0.0001), 5.0- (p < 0.0001) and 1.9-fold (p < 0.0001) greater, respectively in PPARα^-/- ^mice than in PPARα^+/+ ^mice (Figure 1).

**Figure 2:**
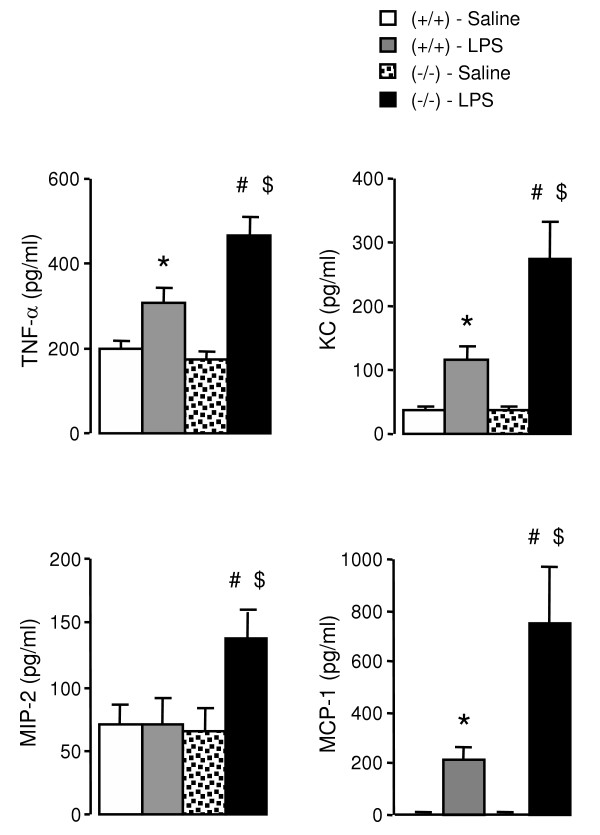
**Chemoattractant levels in BALF from PPARα^+/+ ^(+/+) and PPARα^-/- ^(-/-) mice exposed to LPS or saline**. Data are mean ± SEM of n = 9–12 animals. Statistically significant differences at α = 0.05: (*) when compared to PPARα^+/+ ^mice treated with saline; (#) when compared to PPARα^-/- ^mice treated with saline; and ($) when compared to PPARα^+/+ ^mice treated with LPS.

Cell infiltration induced by LPS was associated with a significant increase in BALF levels of the chemoattractants, TNF-α, KC and MCP-1 in both PPARα^+/+ ^and PPARα^-/- ^mice (Figure 2). These levels were however 1.5- (p = 0.0003), 2.3- (p = 0.0008) and 3.5-fold (p = 0.0012) greater, respectively in PPARα^-/- ^animals when compared to PPARα^+/+ ^mice (Figure 2). PPARα^-/- ^mice exposed to LPS also displayed a significant rise in MIP-2 in BALF (2.0-fold, p = 0.0065), whereas LPS-treated PPARα^+/+ ^animals exhibited no changes in this chemokine.

**Figure 3:**
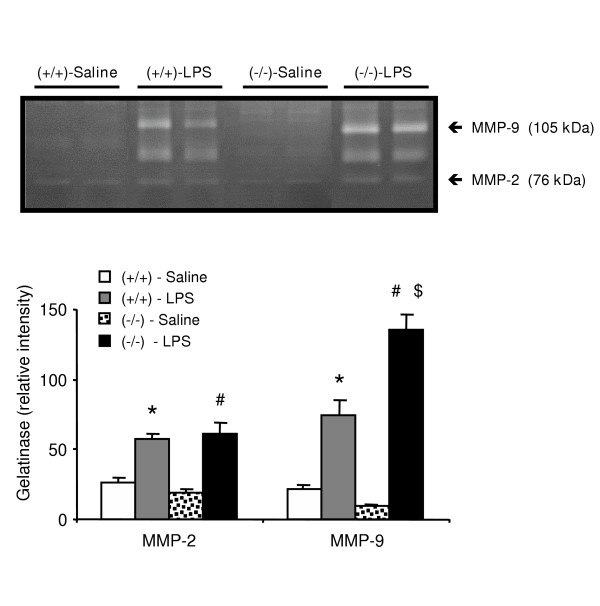
**MMP-2 (76 kDa) and MMP-9 (105 kDa) activity in BALF from PPARα^+/+ ^(+/+) and PPARα^-/- ^(-/-) mice exposed to LPS or saline**. Upper panel shows gelatin zymogram from two representative animals in each group. Lower panel shows data of all animals in each group (n = 10–13) expressed as mean ± SEM. Statistically significant differences at α = 0.05: (*) when compared to PPARα^+/+ ^mice treated with saline; (#) when compared to PPARα^-/- ^mice treated with saline; and ($) when compared to PPARα^+/+ ^mice treated with LPS.

Saline-exposed PPARα^-/- ^mice exhibited similar low MMP-2 (76 kDa) and MMP-9 (105 kDa) activity in BALF when compared to saline-exposed PPARα^+/+ ^animals (Figure 3). Upon exposure to LPS, PPARα^+/+ ^and PPARα^-/- ^mice displayed a significant increase in both MMP-2 and MMP-9 activity, when compared to animals exposed to saline (Figure 3). MMP-2 levels were similar in LPS-treated PPARα^-/- ^and PPARα^+/+ ^mice (61 ± 8 *vs *58 ± 4). In contrast, MMP-9 levels were 1.8-fold (p < 0.0001) greater in PPARα^-/- ^animals than in PPARα^+/+ ^mice.

### Reduced cell infiltration, chemoattractant production and MMP activity in wild-type mice upon PPARα activation by fenofibrate

**Figure 4:**
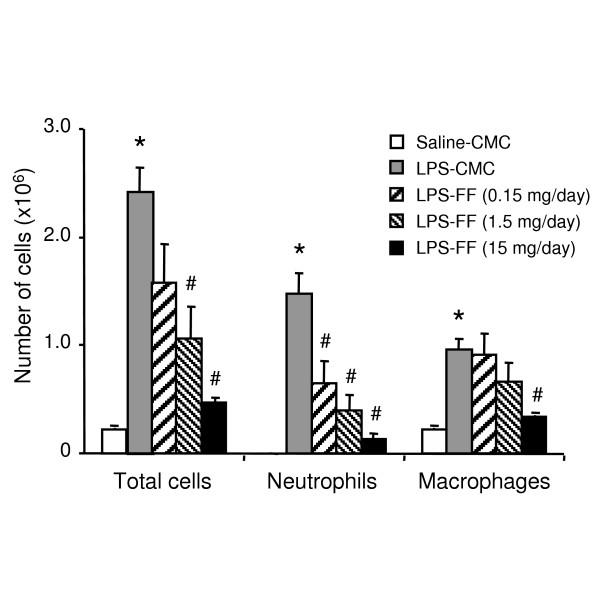
**Dose-dependent reduction of cell infiltration in wild-type mice exposed to LPS upon PPARα activation by fenofibrate**. Number of total cells, neutrophils and macrophages in BALF from C57BL/6 mice exposed to LPS and treated with increasing doses of fenofibrate (0.15 to 15 mg/day) or its vehicle (1% CMC), when compared to mice exposed to saline and treated with CMC. Data are mean ± SEM of n = 6 animals. Statistically significant differences at α = 0.05: (*) when compared to mice exposed to saline and treated with CMC; (#) when compared to mice exposed to LPS and treated with CMC.

Exposure to LPS resulted in marked increases in total cell, neutrophil and macrophage number in BALF from C57BL/6 mice (Figure 4). These increases were dose-dependently reduced by fenofibrate (0.15 to 15 mg/day). Reduction in total cell, neutrophil and macrophage number reached 80% (p < 0.0001), 91% (p < 0.0001) and 64% (p < 0.0001), respectively in BALF from mice treated with 15 mg/kg of the PPARα activator when compared to mice treated with the vehicle, CMC (Figure 4). Fenofibrate (15 mg/day) inhibited also total cell (p = 0.0055), neutrophil (p < 0.0001) and macrophage (p = 0.0064) infiltrate induced by LPS in PPARα^+/+ ^mice (Table 1). In contrast, LPS-exposed PPARα^-/- ^mice treated with 15 mg/day fenofibrate failed to exhibit changes in inflammatory cell infiltrate, demonstrating that PPARα mediates the anti-inflammatory activity of fenofibrate (Table 1).

**Table 1:** Cell infiltration in LPS-exposed PPARα^+/+ ^and PPARα^-/- ^mice treated with fenofibrate.

**Group**	**Number of cells (×10^6^)**		
	**Total**	**Neutrophils**	**Macrophages**

(+/+)-LPS-CMC	1.93 ± 0.11	0.95 ± 0.11	0.98 ± 0.13
(+/+)-LPS-FF	0.73 ± 0.08 (*)	0.23 ± 0.07 (*)	0.50 ± 0.05 (*)
(-/-)-LPS-CMC	3.46 ± 0.38 (*)	1.85 ± 0.28 (*)	1.60 ± 0.25 (*)
(-/-)-LPS-FF	3.13 ± 0.54 (n.s.)	1.73 ± 0.30 (n.s.)	1.39 ± 0.28 (n.s.)

Data are mean ± SEM of n = 6–8 animals. (*): statistically significant differences at α = 0.05 when compared to PPARα^+/+ ^mice treated with CMC. (n.s.): non statistically different when compared to PPARα^-/- ^mice treated with CMC.

**Figure 5:**
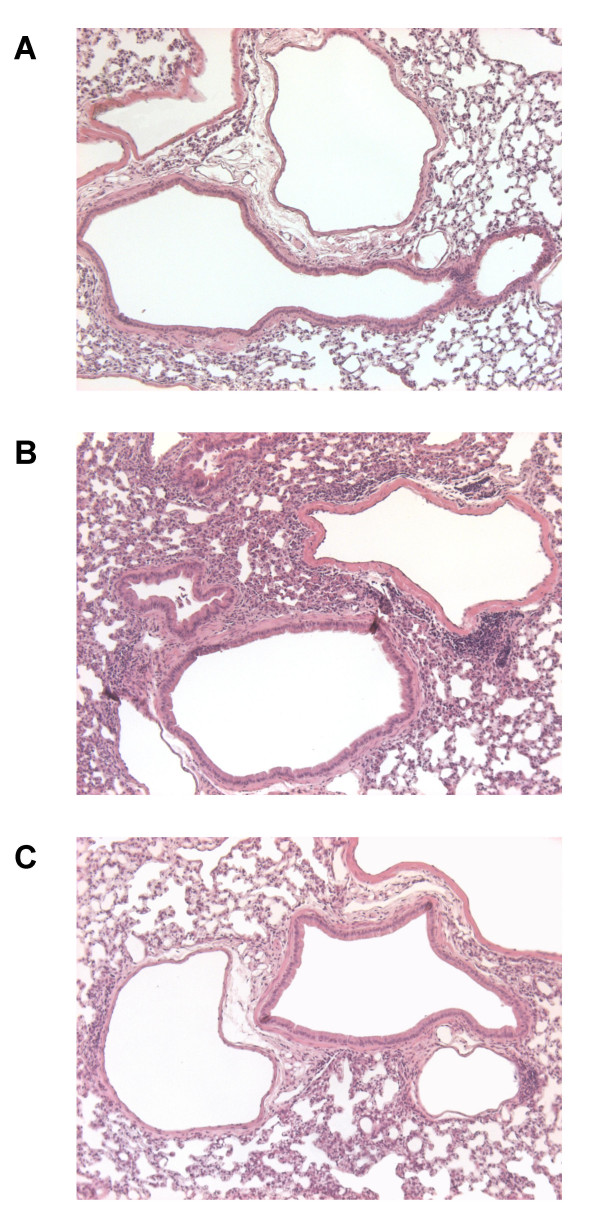
**Histological analysis of lung tissue from wild-type mice**. Lung sections showing a massive inflammatory cell infiltrate in perivascular and alveolar tissue of C57BL/6 mice exposed to LPS and treated with CMC (B), when compared to mice exposed to saline (A). Reduced cell infiltrate in lung tissue from mice exposed to LPS and treated with fenofibrate (C).

Histological examination of lung tissue confirmed the anti-inflammatory effect of fenofibrate. Indeed, whereas a massive inflammatory cell infiltration was observed in perivascular and alveolar tissue of C57BL/6 mice exposed to LPS and treated with CMC when compared to mice exposed to saline (Figure 5A et 5B), a marked reduction in cell infiltration was observed on lung sections from mice exposed to LPS and treated with fenofibrate (Figure 5C).

**Figure 6:**
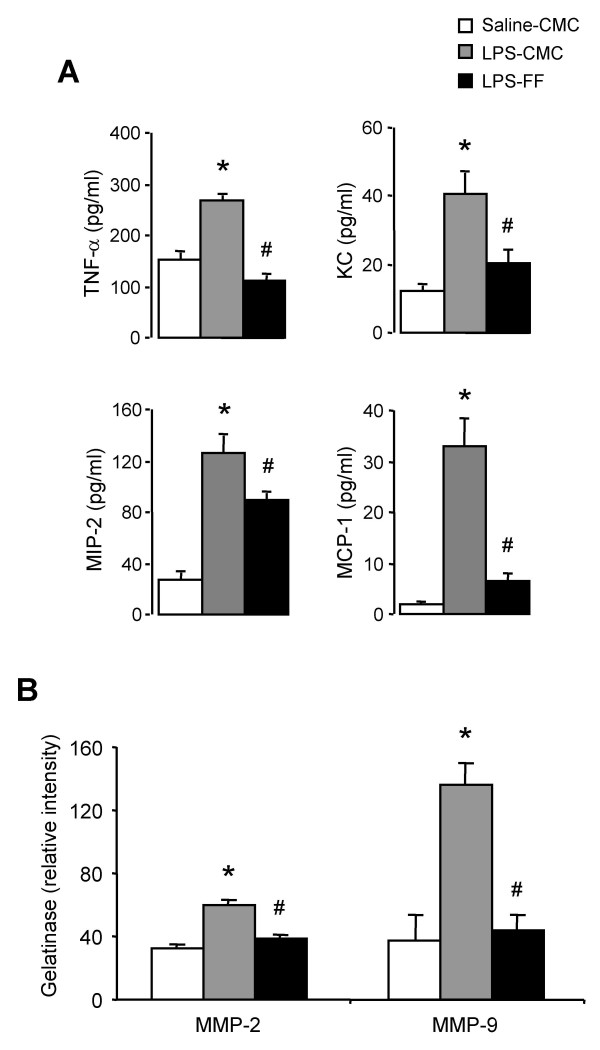
**Reduced chemoattractant production and MMP activity in wild-type mice upon PPARα activation by fenofibrate**. Chemoattractant levels (A) and MMP-2 and MMP-9 activity (B) in BALF from C57BL/6 mice exposed to LPS and treated with fenofibrate (15 mg/day, black bars) or its vehicle (1% CMC, grey bars), when compared to mice exposed to saline and treated with CMC (open bars). Data are mean ± SEM of n = 7–8 animals. Statistically significant differences at α = 0.05: (*) when compared to mice exposed to saline and treated with CMC; (#) when compared to mice exposed to LPS and treated with CMC.

C57BL/6 mice exposed to LPS and treated with CMC displayed also increases in TNF-α, KC, MIP-2 and MCP-1 in BALF when compared to saline-exposed mice (Figure 6A). Treatment with fenofibrate (15 mg/day) inhibited these increases by 59% (p < 0.0001), 50% (p = 0.0015), 30% (p = 0.0058) and 69% (p < 0.0001), respectively (Figure 6A).

Treatment with fenofibrate (15 mg/day) also dramatically reduced LPS-induced increase in MMP-2 and MMP-9 activity (Figure 6B). Indeed, whereas MMP-2 and MMP-9 activity was increased by 1.8- (p < 0.0001) and 3.6-fold (p < 0.0001), respectively in BALF from LPS-exposed mice treated with CMC when compared to saline-exposed mice, animals exposed to LPS and treated with fenofibrate displayed MMP levels similar to those measured in saline-exposed animals.

## Discussion

In this study, we have addressed the role of PPARα in a mouse model of LPS-induced airway inflammation. Using both genetic and pharmacological approaches, our data clearly showed that PPARα downregulates cell infiltration, chemoattractant production and enhanced MMP activity triggered by LPS in mouse lung.

As expected, wild-type mice exposed to LPS exhibited a massive recruitment of inflammatory cells in the airways, composed of neutrophils and macrophages. This cell infiltration was associated with an increase in BALF levels of the pro-inflammatory and chemoattractant cytokine, TNF-α and by a rise in the levels of the CXC chemokines, MIP-2 and KC and of the CC chemokine, MCP-1. Exposure to LPS also induced a marked increase in MMP-2 and MMP-9 activity in BALF, when compared to saline exposure. This model reproduced several features of the inflammatory response observed during acute lung injury or COPD [1,6,13]. Using this model, we found that PPARα^-/- ^mice exposed to LPS displayed enhanced neutrophil and macrophage number in BALF when compared to PPARα^+/+ ^animals, whereas wild-type mice treated with the PPARα activator, fenofibrate exhibited reduced cell infiltrate. Furthermore, we demonstrated fenofibrate selectivity by showing absence of effect of fenofibrate in PPARα^-/- ^animals. Taken together, these results suggest that PPARα activation may have a beneficial effect in airway inflammatory diseases involving neutrophil and monocyte recruitment. In agreement with our results, Birrell et al. recently proposed that agonists of another PPAR receptor, PPARγ may have a therapeutic potential in respiratory diseases involving neutrophilia [29]. Our study adds to these previous findings by showing that PPARα agonists may also be effective in blocking recruitment of monocytes, which play a pivotal role in the pathophysiology of COPD, as well as of pulmonary fibrosis. By contrast, Trifilieff et al. found that PPARα ligands failed to inhibit neutrophil recruitment induced by LPS in BALF from mice [30]. Differences in the mode of exposure to LPS could explain this discrepancy. Indeed, whereas these authors exposed female mice intranasally to a single high dose of LPS (0.3 mg/kg) for a short period of time (3 h), the present study was carried out in male animals using four repeated instillations of a 7.5-fold lower dose of LPS (40 μg/kg). Indeed, these modes of exposure may trigger different inflammatory responses. Likewise, nature (GW 9578 vs fenofibrate) and route of delivery (local vs oral) of PPARα agonists may be another source of discrepancy. Therefore, by both genetic and pharmacological approaches, our data clearly demonstrate that PPARα downregulates neutrophil and monocyte infiltration in mouse lung.

We also found that PPARα^-/- ^mice exposed to LPS displayed increased levels of TNF-α in BALF when compared to PPARα^+/+ ^animals, whereas wild-type mice treated with fenofibrate exhibited reduced TNF-α levels. As a pro-inflammatory cytokine, TNF-α that is released by macrophages or airway epithelial cells upon activation plays an important role in neutrophilic inflammation induced by LPS [4]. Indeed, TNF-α triggers the release of CXC chemokines, such as MIP-2 and KC that are involved in LPS-induced intrapulmonary recruitment of neutrophils [2,3]. As well, MCP-1, which plays a central role in monocyte recruitment to inflamed tissues, is produced by pulmonary macrophages and airway epithelial cells in response to TNF-α or LPS [31,32]. In the present study, release of MIP-2, KC and MCP-1 triggered by LPS instillation was greater in BALF from PPARα^-/- ^mice when compared to PPARα^+/+ ^animals. Conversely, wild-type mice treated with fenofibrate displayed decreased levels of these chemokines when compared to vehicle-treated animals. Taken together, our results suggest that downregulation of TNF-α and of the CXC and C-C chemokines, MIP-2, KC and MCP-1 contributes to PPARα-induced inhibition of neutrophil and macrophage airway recruitment in our model.

PPARα agonists were recently reported to reduce LPS- and IL-1β-induced secretion of MMP-9 in human monocytes and rat mesangial cells, respectively [24,25]. However, the effect of PPARα on MMP production in vivo is so far unknown. In the present study, we demonstrate that PPARα downregulates increase in MMP-2 and MMP-9 activity triggered by LPS in mouse lung. Indeed, whereas PPARα^-/- ^mice displayed a greater increase in MMP activity in BALF upon exposure to LPS when compared to PPARα^+/+ ^animals, wild-type mice exposed to LPS exhibited decreased levels of MMP when treated by fenofibrate. Sources of MMP in the lung are numerous, particularly under inflammatory conditions. Among them, neutrophils and macrophages are considered as the major sources of MMP-9 [11]. Therefore, downregulation of MMP-9 production by PPARα may result from decreased cell infiltration. In neutrophils, MMP-9 is stored in specific granules from which it is readily released, in particular upon stimulation by LPS or chemoattractant factors, like IL-8 [33]. Downregulation of MMP-9 production by PPARα could alternatively result from decreased neutrophil activation. MMP-9 is believed to play a major role in lung remodeling. Indeed, in addition to digestion of extracellular matrix proteins, MMP-9 increases the activity of other proteases, as well as of chemoattractants and growth factors (for review: [34]). By providing evidence that PPARα downregulates MMP activity in vivo, our study reinforces the idea that the nuclear receptor PPARα may play a beneficial role in tissue remodeling.

Several studies have reported that PPARα inhibits the NF-κB pathway, which plays a critical role in LPS signaling as well as in the expression of the chemokines, MIP-2, KC and MCP-1 and of MMP-9 [35]. This property could account for the beneficial effect of PPARα observed in the present study. However, several other mechanisms could be involved. This includes production of anti-inflammatory mediators, such as IL-10. Indeed, fenofibrate was reported to suppress autoimmune myocarditis in rats by stimulating expression of this cytokine [36]. As well, inhibition of cell recruitment could be implicated. Thus, activation of PPARα was reported to inhibit chemotaxis of inflammatory cells, including macrophages [37,38]. Finally, resolution of inflammation through stimulation of inflammatory cell apoptosis may also be involved, since activation of PPARα was shown to induce apoptosis of macrophages [39].

## Conclusion

In conclusion, using both genetic and pharmacological approaches, our study provides evidence that PPARα downregulates neutrophil and monocyte infiltration induced by LPS in mouse lung. Our data also demonstrated that this beneficial effect of PPARα involves downregulation of the production of neutrophil and monocyte chemoattractants, including the CXC and C-C chemokines, MIP-2, KC and MCP-1, and of MMP that play a major role in tissue remodeling. We postulate that PPARα agonists, and in particular fenofibrate may have a therapeutic potential in airway inflammatory disorders involving neutrophil and monocyte, such as acute lung injury and COPD.

## List of abbreviations

BALF: bronchoalveolar lavage fluid

CMC: carboxylmethylcellulose

COPD: chronic obstructive pulmonary disease

EDTA: ethylenediaminetetraacetic acid

IL: interleukin

KC: keratinocyte derived-chemokine

LPS: lipopolysaccharide

MIP-2: macrophage inflammatory protein-2

MMP: matrix metalloproteinase

PPAR: peroxisome proliferator-actived receptor

MCP-1: monocyte chemoattractant protein-1

TNF-α: tumor necrosis factor-α

## Authors' contributions

CDO, JB and IG have made substantial contributions to acquisition and analysis of data.

CDO, VL and FP have made substantial contributions to conception and design of the study.

CDO and FP have been involved in drafting the article.

JA, NF and VL have been involved in revising the article critically for important intellectual content.
